# Post-mortem findings in *Staphylococcus aureus* acute infective endocarditis

**DOI:** 10.4322/acr.2020.212

**Published:** 2020-11-20

**Authors:** Deepika Phogat, Mukul Bajpai, Prosenjit Ganguli, Vimal Upreti

**Affiliations:** 1 151 Base Hospital, Department of Pathology, Guwahati, Assam, India; 2 Command Hospital Eastern Command, Department of Pathology, Kolkata, West Bengal, India; 3 151 Base Hospital, Department of Internal Medicine, Guwahati, Assam, India

**Keywords:** Staphylococcus aureus, Staphylococcal Infections, Endocarditis, Bacterial, Sudden death

## Abstract

Infective endocarditis (IE) is a microbial infection of the heart valves or the mural endocardium that leads to the formation of vegetations composed of thrombotic debris and microorganisms often associated with the destruction of the cardiac tissues. Most of the infections are bacterial (bacterial endocarditis), although fungi and other microorganisms can be etiological agents. Causative organisms differ among the major high-risk groups. Virulent microorganisms like *Staphylococcus aureus,* commonly found on the skin, can infect normal or deformed valves and are responsible for 20-30% of all IE cases. *Staphylococcus aureus* is the major offender in IE among intravenous drug abusers. Acute infective endocarditis is typically caused by infection of a previously normal heart valve by a highly virulent organism (e.g., *Staphylococcus aureus*) that rapidly produces necrotizing and destructive lesions. These infections may be difficult to cure with antibiotics, and despite appropriate treatment, death can ensue within days to weeks. Here we present autopsy findings of a 31-year-old male patient who died of acute infective endocarditis caused by *Staphylococcus aureus* as the causative organism.

## INTRODUCTION

Infective endocarditis (IE) is an entity that affects the endocardium and heart valves with thrombotic debris and organisms-forming vegetations, associated with the destruction of the cardiac tissues.

Prompt diagnosis, identification of the offending agent, and effective treatment of IE are important in limiting morbidity and mortality.

Etiological infective agents involve bacteria (most commonly) along with fungi. These infectious agents differ in virulence and vary according to various high-risk groups. *Staphylococcus aureus* is a virulent organism that can infect normal valves and is the major offender in IE among intravenous drug abusers.^1^

Other bacterial causes include enterococci and so-called HACEK group (Haemophilus, Actinobacillus, Cardiobacterium, Eikenella, and Kingella) commensals in the oral cavity.

Prosthetic valve endocarditis is caused most commonly by coagulase-negative staphylococci (e.g., *Staphylococcus epidermidis*).

Other agents causing endocarditis include gram-negative bacilli and fungi.

In about 10% of endocarditis cases, no organism can be isolated from the blood (culture-negative endocarditis). Reasons include prior antibiotic therapy, difficulties in isolating the offending agent, or because deeply embedded organisms within the enlarging vegetation are not released into the bloodstream.

Acute infective endocarditis is typically caused by infection of a previously normal heart valve by a highly virulent organism (e.g., *Staphylococcus aureus*) that rapidly produces necrotizing and destructive lesions. These infections may be difficult to cure with antibiotics alone and usually require surgery. Despite appropriate treatment, death can ensue within days to weeks.^1^

Here we present autopsy findings of a 31-year old male patient who died of a *Staphylococcus aureus* acute infective endocarditis.

## CASE REPORT

A 31-year-old male was referred to the hospital with breathlessness and altered sensorium, where he was admitted with a working diagnosis of alcohol-related cardiomyopathy and acute kidney injury. He was known to be an alcohol abuser. However, the information on any other drug abuse was unknown. Clinically he was stuporous, with a temperature of 38.3^o^ C, pulse 110/min, blood pressure of 100/70 mmHg, and room-air oximetry was 98%. The physical examination showed generalized puffiness, ecchymosis over right arm and leg, icterus, and raised jugular vein pressure. Chest auscultation revealed bilateral basal crepitations. The abdominal examination showed hepatomegaly. The clinical diagnosis was chronic liver disease, hepatic encephalopathy, coagulopathy, and sepsis with multiple organ dysfunction syndrome. Therapeutic attempts with IV antibiotics and vasoactive drugs failed to improve the clinical status, and the patient passed away 6 hours after admission.

On investigation, hemoglobin was 10.8g/dL (reference range [RR];13-17g/dl) with normocytic and normochromic hematimetric indices; white blood count of 30,000/mm^3^ (RR; 4000-11000/mm^3^), with polymorphonuclear leukocytes of 92%, lymphocytes 02%, monocytes 04%, and eosinophils 02%. The platelet count was 11,000/mm^3^ (RR:1.5-4.0x 10^5^/mm^3^). INR was 1.3 (RR; 0.8-1.2). Renal function tests showed urea of 257mg/dL (RR;15-40mg/dl) and creatinine of 4.2mg/dL (RR;0.55-1.3mg/dl). Bilirubin was raised, 8.4mg/dL (RR;0.2-1mg/dl); with direct and indirect bilirubin 6.7mg/dL (RR:0.1-0.3mg/dl) and 1.7mg/dl (RR; 0.1-0.7mg/dl) respectively; ALT of 224 IU/L (RR; 15-37IU/L) and AST of 102 IU/L (RR; 12-78IU/L). C-Reactive Protein was 295mg/dL (RR; 0.0-0.5mg/dl); D-dimer was 9039.33 (RR: 50-500ng/mL). Viral markers: HIV, HBsAg, Anti HCV were negative.

The autopsy was performed with the working diagnosis of sepsis with multiple organ dysfunction syndrome with pre-existing alcohol-related cardiomyopathy and chronic liver disease.

## AUTOPSY FINDINGS

On external examination, the corpse was averagely built and nourished with the length of 168cm and the weight of 74kg. Puffiness of the skin, rigor mortis, and postmortem lividity were present. Skin showed multiple venipuncture marks on the right and left arm. Multiple tattoos were noted over both upper limbs and multiple ecchymotic patches seen over the right arm, right leg, abdomen and chest. There was bleeding from nostrils and mouth.

**Figure 1:**
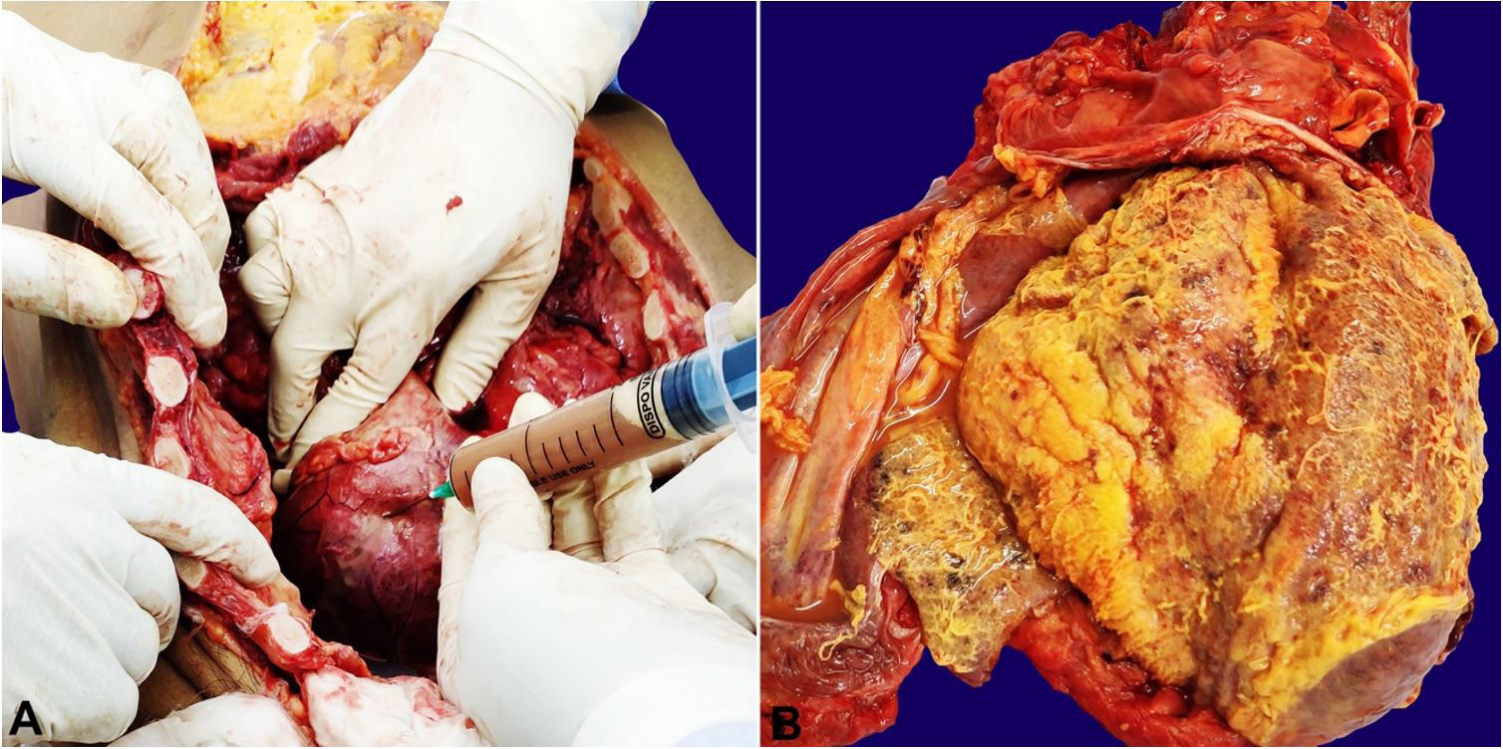
Gross view of the: **A** – Opened thoracic cavity with pericardial sac puncture. Note the purulent fluid being drained; **B** – Gross view of the heart -purulent pericarditis.

**Figure 2:**
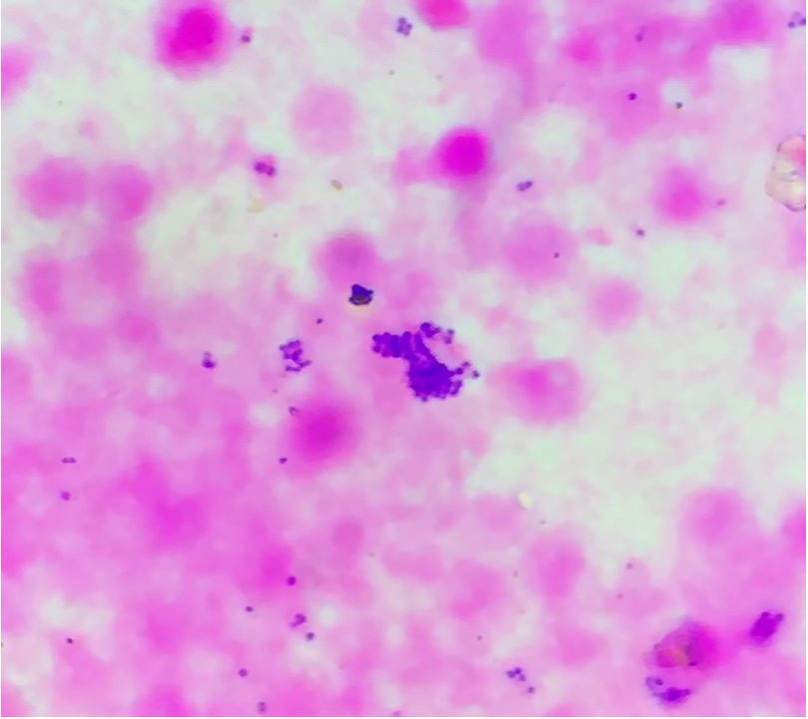
- Pericardial fluid showing gram-positive cocci - *Staphylococcus aureus* identified on the bacteriological study (Gram stain, 40x).

After the thoracic overture, 90 ml of purulent pericardial fluid was drained from the pericardial cavity (Figure 1A). The pericardial surface showed a purulent exudate (Figure 1B) with Gram-positive clustered cocci (Figure 2). The heart was grossly enlarged, weighing 550g (mean RR; 320 g) with left ventricle wall thicknesses of 1.5 cm (RR: 0.5-0.9cm), and the right ventricle wall thickness of 0.8 cm (RR: 0.4-0.7cm).

**Figure 3:**
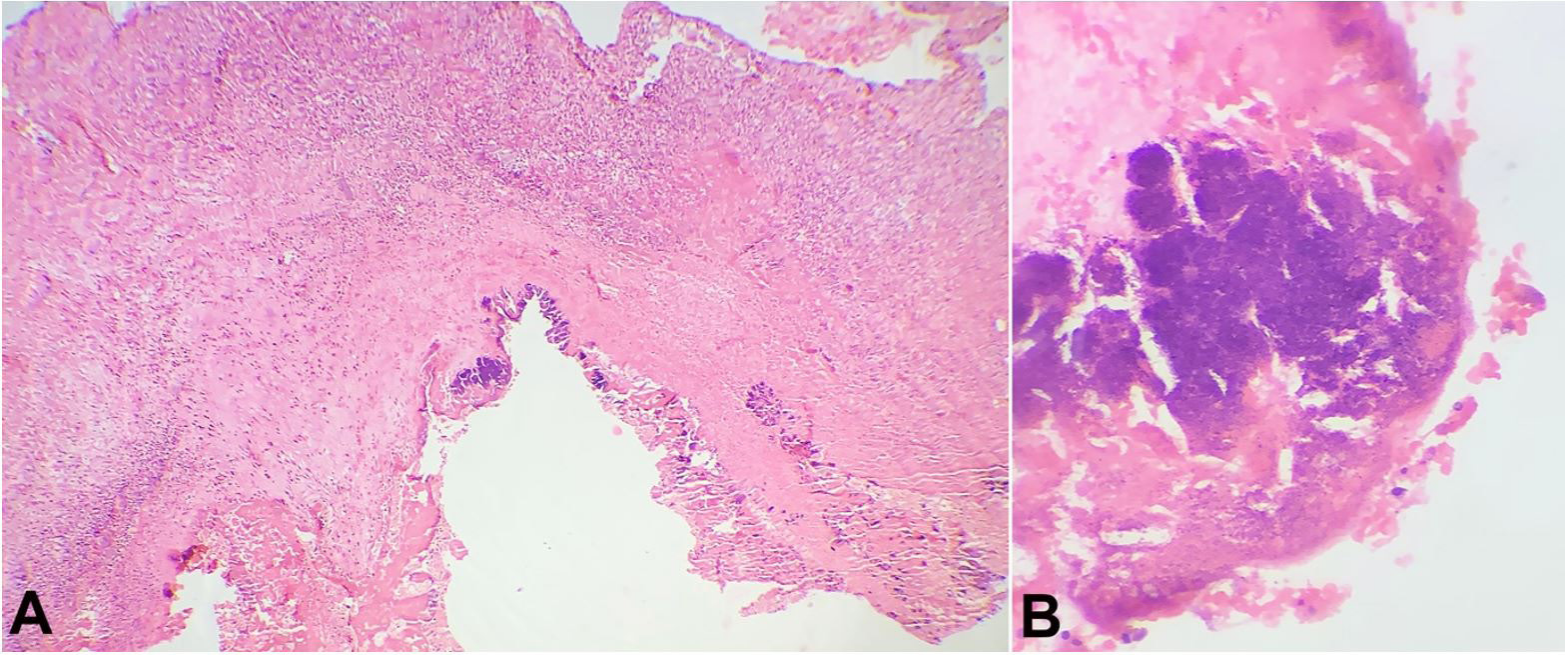
Photomicrograph of the mitral valve. **A** – shows the vegetation with bacterial colonies (H&E stain, 10x); **B** – shows a detail of the vegetation with the bacterial colonies (H&E stain, 40x).

**Figure 4:**
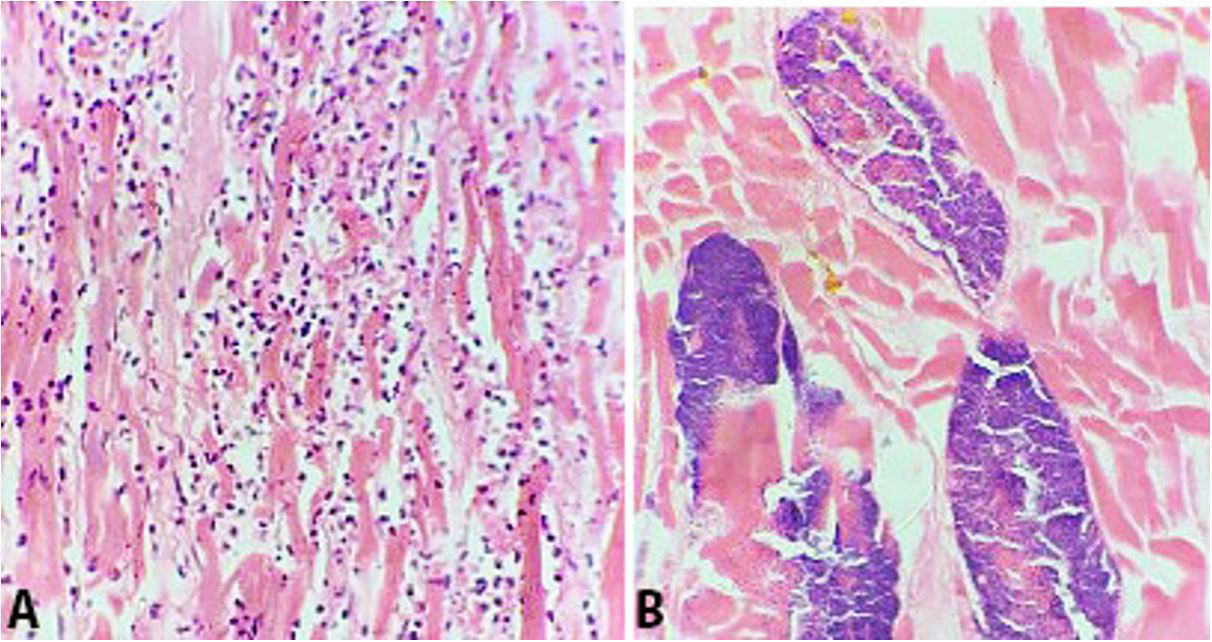
**A** and **B** Photomicrographs of myocardial abscess in left ventricular wall showing neutrophilic infiltrate in the cardiac muscles (**A**) and bacterial colonies in the myocardium (**B**) (H&E, 40x).

The mitral valve showed a small vegetation measuring 0.3 cm in its longest axis. Tricuspid, aortic and pulmonary valves were normal. A para-valvular abscess was found involving the left ventricle wall adjoining the mitral valve. Left and right ventricle walls showed hard whitish areas. On histopathological examination, the mitral valve showed fibrin thrombi and clustered colonies of gram-positive cocci (Figures 3A and 3B). The pericardium showed dense neutrophilic infiltrate, whilst the left ventricle myocardium had focal abscesses along with numerous bacterial colonies in the form of clustered cocci. (Figures 4A and 4B). No features of myocardial fibrosis/hypertrophy of cardiac muscles were identified.

**Figure 5:**
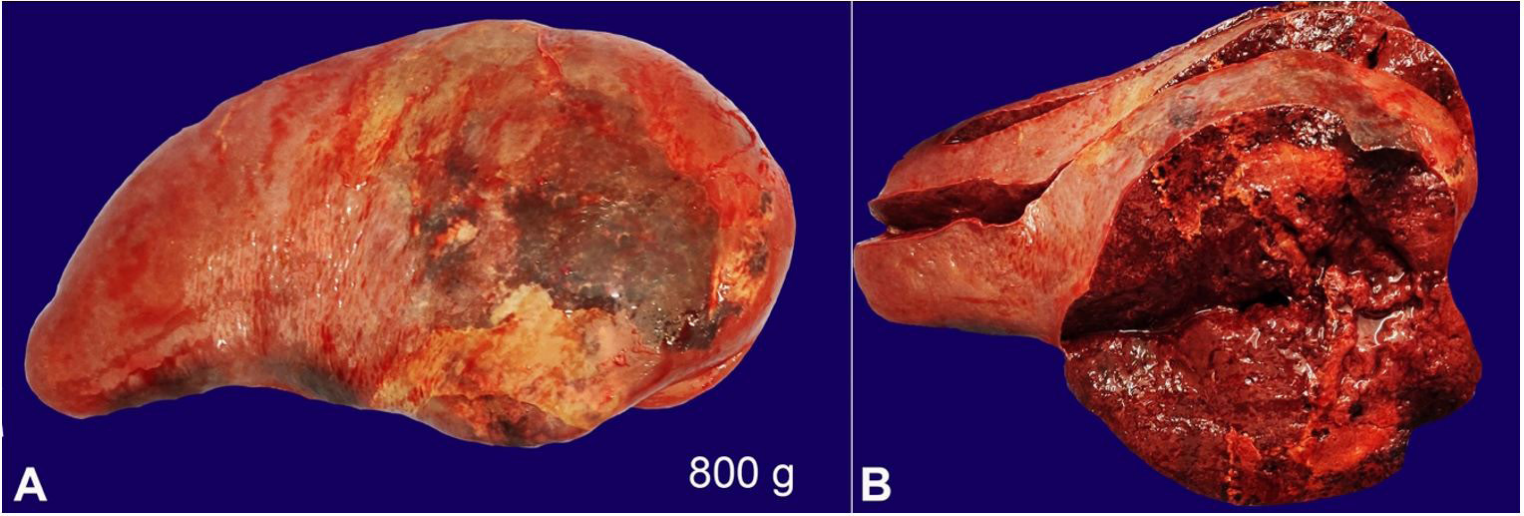
**A** – Gross view of the spleen. Note pale areas indicating septic infarcts over the external surface (weight reference range 112 g); **B** – Cut surface showing septic infarcts and acute splenitis.

**Figure 6:**
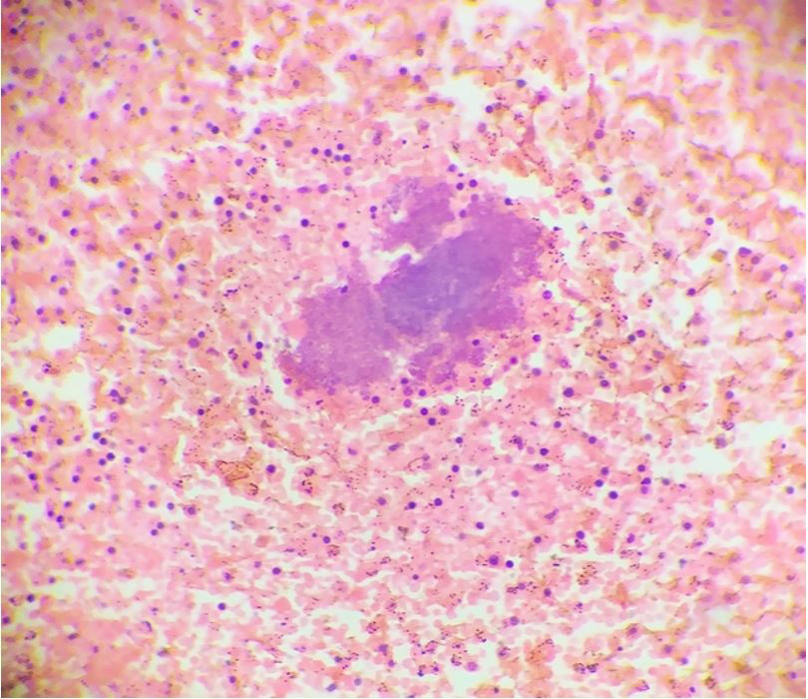
Photomicrograph of the spleen showing numerous bacterial colonies identified as Staphylococcus aureus on bacteriological examination (H&E Stain, 10x).

The lungs were boggy and heavy. The left lung weighed 750 g (mean RR: 375 g),while the right lung weighed 800 g (mean RR: 450 g). The pleural surface was of normal luster with no adhesions between both pleural layers. No consolidation, cavitation, abscess, or mass lesions were seen. The cut surfaces showed oozing blood. Sections from both the lungs showed pulmonary edema and congested capillaries. The liver was enlarged, weighing 2800 g (mean RR:1500 g). The external surface was congested, and the cut surface was unremarkable. Liver histology revealed steatosis, and mild periportal inflammatory infiltrate. No granulomas, necrosis, cirrhosis, or cholestasis were found. Both the kidneys were normal in size. Right kidney weighed 105 g (mean RR 110g), and the left kidney weighed 115 g (mean RR 110 g). The external surface of both the kidneys was smooth and lobulated. No petechiae, infarcts, or scars were seen, and capsules could be easily stripped off. The cut surface of both kidneys showed normal cortico-medullary differentiation. No areas of infarct, hemorrhage, or necrosis were seen. The histological examination of both kidneys revealed normal glomeruli, vessels, and interstitium. Focal tubular epithelial necrosis was seen with desquamation of cells into the tubular lumina was noted. The spleen was enlarged and congested and weighed 800 g (mean RR: 112 g). The external surface showed multiple intermingled pale and congested areas (Figure 5A). The cut surface showed septic infarcts as multiple yellowish areas with necrotic slough (Figure 5B). The spleen histologic examination showed multiple abscesses with dense neutrophilic infiltrates, necrosis, and bacterial colonies (Figure 6).

The external and cut surface of adrenal glands were congested, which on histology, showed areas of hemorrhage. No infarcts were identified on gross as well as histological examination.

## POSTMORTEM MICROBIOLOGY

The samples of postmortem pericardial fluid, heart blood, and ascitic fluid were collected into blood culture bottles and were analyzed with an automated blood culture system (Biomerieux BacT Alert 3D/60). The growth obtained was then sown on to the culture plates and streaked onto slides following which they were fixed and stained with gram stain. Gram-positive growth was identified, and the sample was further analyzed with Fully automated identification and antibiotic sensitivity testing system (Biomerieux Vitek 2 Compact). The analysis revealed methicillin-sensitive *Staphylococcus aureus* as the causative organism.

The cause of death based on the clinical, investigation, and autopsy findings was septicemia caused by *Staphylococcus aureus* and multiple organ failure secondary to infective endocarditis complicated by a myocardial abscess, purulent pericarditis, and multiple splenic abscesses, in a patient addicted to alcohol.

## DISCUSSION

Clinically, acute endocarditis has a stormy onset with rapidly developing fever, chills, weakness, and lassitude. Although fever is the most consistent sign of IE, it can be slight or absent, particularly in older adults, when the only manifestation may be non-specific fatigue, weight loss, and a flu-like syndrome. The diagnosis of IE still relies on the Dukes criteria.^2^ In turn, the pathology criteria involve the demonstration of microorganisms by culture or histologic examination of the valvular vegetation, embolus from vegetation, or intracardiac abscess, as was seen in our case. Major clinical criteria are blood culture(s) positive for a characteristic organism or persistently positive for an unusual organism. Our case had a blood culture positive for *Staphylococcus aureus*.

Minor criteria are predisposing heart lesion or IV drug abuse, fever, lesions including petechiae, subungual/splinter hemorrhages, emboli, septic infarcts, mycotic aneurysm, intracranial hemorrhage, Janeway lesions, immunologic phenomena including glomerulonephritis, Osler’s nodes, Roth spots, and presence of rheumatoid factor.

IE has been classified into acute and subacute forms. Our case was acute infective endocarditis with a fulminant course that led to the patient’s death within a few hours of admission despite active therapeutic attempts. These infections may be difficult to cure with antibiotics alone and usually require surgery.

In contrast, subacute IE is characterized by organisms with lower virulence (viridans-group streptococci) that cause insidious infections of deformed valves with overall less destruction. In such cases, the disease may pursue a protracted course of weeks to months, and cure can usually be achieved with antibiotics.

Our case had purulent pericarditis and cardiac tamponade, which are rare complications of bacterial endocarditis seen in 1.23%-2.5% of cases.^3^ In a study by Katz et al.^4^, cardiac tamponade was noted more commonly in patients with history of alcohol abuse and patients having pre-existing diabetes mellitus. This complication is associated with increased mortality.

In our case, the heart was grossly enlarged with increased right and left ventricle wall thickness. Myocardial inflammation was noted in the left ventricle wall with focal abscesses along with numerous bacterial colonies in the form of clustered cocci; however, no features of myocardial fibrosis or hypertrophy of cardiac muscles were identified. Myocardial inflammation is seen in 40-80% of cases of bacterial endocarditis.^5^

Complications related to embolization are seen in 22-50% of cases that were not seen in our case.^6^

The identification of the predisposing factors involved in the development of infective endocarditis is not that easy. However, intravenous drug abuse, prosthetic heart valves and structural heart disease are common predisposing factors.^7^ ^,^ ^8^

Procedures that involve a breach in the skin like body piercings and body art in the form of tattooing may introduce pathogenic bacteria into the bloodstream, especially when performed without aseptic/sterile techniques. Local infections such as necrotizing fasciitis, cellulitis, and septicemia with *Pseudomonas aeruginosa* and *Streptococcus pyogenes* have been reported in the literature.^9^ ^,^ ^10^ In 2005, the Centers for Disease Control and Prevention (USA), reported 44 cases of community-acquired soft tissue infections with *Staphylococcus aureus* secondary to tattooing, performed by 13 unlicensed body art artists who did not follow sterile techniques.^11^ There have been case reports showing association of body piercing with *Staphylococcus aureus* related IE.^12^ ^,^ ^13^

*Staphylococcus aureus* IE has also been associated with tattooing in patients with and without pre-existing structural cardiac anomalies.^14^ ^,^ ^15^

In our case, skin needling done during tattooing, the possibility of IV drug abuse, and history of alcohol abuse could have contributed to the development of *Staphylococcus aureus* related infective endocarditis. Alcoholism is an important, but neglected factor predisposing to infective endocarditis as studied by Buchbinder et al.^16^ Chronic liver disease also has a significant impact on the prognosis in patients with infective endocarditis and these should, therefore, be considered high risk group.^17^

To conclude, though not reported commonly and not considered a common predisposing factor for IE, skin tattooing done under unsterile techniques and history of alcohol abuse could have a role in *Staphylococcus aureus*-related IE.
